# Fermented Black Soybean and Dehulled Adlay Improve Metabolic Syndrome via AMPK–SIRT1 Activation and Gut Microbiota Modulation

**DOI:** 10.1021/acs.jafc.5c15101

**Published:** 2026-02-23

**Authors:** Ya-Ru Kuo, Yi-Wei Zheng, Pin-Yu Ho, Yi-Chen Lo, Po-Jung Tsai, Min-Hsiung Pan

**Affiliations:** † Institute of Food Sciences and Technology, 33561 National Taiwan University, Taipei City 10617, Taiwan; ‡ Graduate Program of Nutrition Science, School of Life Science, 34879 National Taiwan Normal University, Taipei City 10610, Taiwan; § Department of Medical Research, China Medical University Hospital, China Medical University, Taichung City 40402, Taiwan; ∥ Department of Health and Nutrition Biotechnology, Asia University, Taichung City 41354, Taiwan

**Keywords:** metabolic syndrome, insulin resistance, Bacillus subtilis *natto*, gut microbiota, obesity

## Abstract

This study investigated the metabolic efficacy of*Bacillus subtilis*-fermented black soybean and dehulled adlay (FBA) compared to its unfermented black soybean and dehulled adlay (UFBA) in high-fat, high-fructose-diet (HFFD)-fed C57BL/6J mice. FBA supplementation exhibited superior effects in attenuating body weight gain, visceral adiposity, dyslipidemia, and hepatic steatosis while improving insulin sensitivity. These benefits were consistent with the modulation of hepatic AMPK–SIRT1 and PI3K–Akt signaling pathways, suggesting enhanced lipid oxidation and suppressed lipogenesis. Furthermore, FBA restructured the gut microbiota by reversing HFFD-induced dysbiosis, notably decreasing the Firmicutes-to-Bacteroidetes (F/B) ratio and specifically elevating cecal propionate levels. These findings demonstrate that fermentation significantly potentiates the bioactivity of the substrate, suggesting FBA as a potential dietary strategy that may attenuate metabolic syndrome (MetS) features.

## Graphical abstract

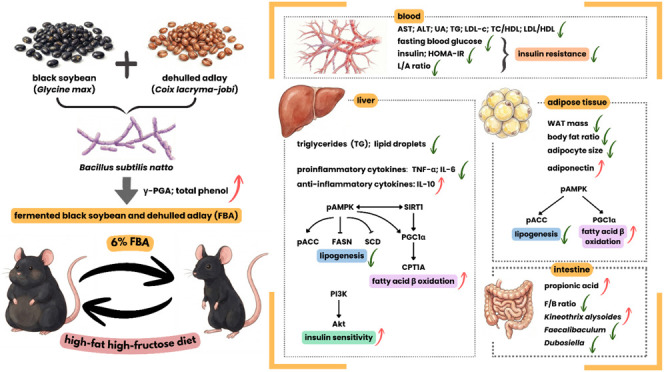

## 1 Introduction

Metabolic syndrome (MetS) is commonly recognized as a complex condition characterized by abdominal obesity, insulin resistance, hypertension, and dyslipidemia. Initially predominant in Western societies, MetS has evolved into a global health crisis due to the widespread adoption of Western lifestyles.^1^ Currently, MetS affects approximately 34% of adults globally, with its incidence continuing to rise steadily.^2^ Although the precise pathophysiology of MetS remains incompletely understood, several key factors contribute to its development, including genetic predisposition, high-calorie diets, and physical inactivity, all of which significantly affect metabolic dysfunction, particularly insulin resistance. Consumption of high-fat diets leads to excessive lipid accumulation, impairing both fat oxidation and energy expenditure and ultimately resulting in obesity and systemic metabolic disturbances. Moreover, hypertrophied adipocytes release free fatty acids (FFA), which in turn trigger hepatic lipid accumulation and metabolic dysregulation.^3^ At the molecular level, AMP-activated protein kinase (AMPK) and Sirtuin-1 (SIRT1) are crucial regulators of energy balance, controlling glucose and lipid metabolism. Activation of AMPK increases NAD^+^ levels, which in turn activate SIRT1. Conversely, SIRT1 deacetylates liver kinase B1 (LKB1), facilitating AMPK phosphorylation.^4^ However, excessive nutrient intake, especially from high-fat diets, disrupts this regulatory loop, causing lipid metabolism disorders, inflammation, and insulin resistance. In addition to lipid overload, fructose, frequently consumed in modern diets, accelerates metabolic dysfunction by bypassing glycolytic regulation, enhancing hepatic de novo lipogenesis, and increasing oxidative stress. Excessive fructose intake results in hepatic lipid accumulation, insulin resistance, and systemic inflammation.^5,6^ Long-term consumption of high-fat, high-fructose diets (HFFD) reliably induces MetS-like symptoms in animal models, making it a relevant model for study.^7,8^ Gut microbiota composition is also closely associated with metabolic health.^9^ High-fat dietary patterns can disturb microbial balance (dysbiosis), trigger inflammation, and exacerbate MetS-related conditions.^10–12^ Therefore, dietary interventions that target both host metabolic pathways and gut microbiota offer a promising therapeutic strategy.

Black soybeans (*Glycine max*) and dehulled adlay (*Coix lacryma-jobi*) are rich sources of bioactive compounds with antiobesity and anti-inflammatory properties. Black soybean polyphenols, anthocyanins, and isoflavones are known to enhance lipid metabolism and insulin sensitivity,^13–15^ while soybean protein may positively modulate gut microbiota.^16,17^ Similarly, dehulled adlay, abundant in coixenolide, coixan, and unsaturated fatty acids (oleic and linoleic acid), effectively regulates glucose metabolism and alleviates oxidative stress.^18–20^ Furthermore, γ-Polyglutamic acid (γ-PGA), a polymer produced by*Bacillus subtilis* fermentation, exerts multifaceted metabolic benefits, including lipid regulation and increased short-chain fatty acid (SCFA) production.^21–23^ Our previous research demonstrated that cofermented black soybean and dehulled adlay (FBA) mitigates aging-related physiological decline and modulates gut microbiota.^24^ Given that aging and metabolic syndrome share key pathophysiological features, including chronic low-grade inflammation, oxidative stress, and gut dysbiosis. We hypothesized that FBA may also be effective in ameliorating MetS. Specifically, FBA’s ability to reduce senescence markers and inflammatory cytokines in aged mice suggests it may influence upstream regulators of metabolic homeostasis relevant to obesity and insulin resistance.

This study examined the potential of FBA and unfermented black soybean and dehulled adlay (UFBA) to mitigate metabolic disorders induced by a 15-week HFFD regimen in C57BL/6J mice. We comprehensively assessed the impact of these interventions on obesity development, lipid metabolism, insulin resistance, inflammatory markers, and gut microbiota composition. These findings aim to elucidate the efficacy of FBA as a functional food ingredient for enhancing metabolic health.

## 2 Materials and Methods

### 2.1 Chemicals and Antibodies

The study used the following chemicals and reagents: acrylamide (AMRESCO, Solon, OH), Bio-Rad Protein Assay Dye (Bio-Rad Laboratories, Hercules, CA), tetramethylethylenediamine (TEMED) (Bioshop, Burlington, ON, Canada), ammonium persulfate (APS), bovine serum albumin (BSA), and formalin (all from Sigma-Aldrich, St. Louis, MO). For histological procedures, the following materials were obtained: eosin stain, hematoxylin, and paraffin from Leica (Leica Biosystems, Nussloch, Germany). The primary antibodies were sourced from multiple manufacturers: acetyl-CoA carboxylase (ACC) and pACC were obtained from Santa Cruz Biotechnology (Dallas, TX); AMPK, pAMPK, phosphoinositide 3-kinase (PI3K), pPI3K, Akt, and pAkt from Cell Signaling Technology (Beverly, MA); SIRT1, fatty acid synthase (FASN), peroxisome proliferator-activated receptor γ coactivator-1α (PGC-1α), and carnitine palmitoyltransferase 1A (CPT1A) from Proteintech (IL); stearoyl-CoA desaturase (SCD) from ABclonal (Wuhan, China); and adiponectin and GAPDH from Abcam (Cambridge, U.K.). The HRP-conjugated secondary antibodies, including Goat Anti-Rabbit IgG (GTX213110-01) and Goat Anti-Mouse IgG (GTX213111-01), were purchased from GeneTex (Irvine, CA).

### 2.2 Sample Preparation

The FBA was produced using specific cultivars: Tainan No. 3 black soybean and Taichung Selection No. 5 dehulled adlay were both obtained from the Taiwan Farmers’ Cooperative. The*B. subtilis* *natto* strain (YCL-L-039) used in the fermentation process was provided by Dr. Lo’s laboratory at National Taiwan University. Based on a modified protocol adapted from a previous study,^24^ the preparation involved steaming and sterilization steps, followed by inoculation with *B. subtilis* *natto* at a concentration of 10^6^ CFU/g and subsequent fermentation under controlled conditions.

### 2.3 Animal Experiments

This study received approval from the Institutional Animal Care and Use Committee (IACUC Approval No. NTU112-EL-00022). Thirty-two male C57BL/6J mice, aged 4 weeks, were sourced from the National Laboratory Animal Center (Taipei, Taiwan) and maintained in controlled conditions (22 ± 2 °C, 50 ± 10% humidity) with a 12-h light/dark cycle. Following a 1-week acclimation period, the mice were randomly divided into four groups (*n* = 8) for a 15-week feeding trial: ND (normal diet, LabDiet 5001), HFFD (high-fat, high-fructose diet, D12451 with 20% fructose water), UFBA (HFFD supplemented with 6% unfermented black soybean and dehulled adlay), and FBA (HFFD supplemented with 6% fermented black soybean and dehulled adlay). The dosage of the supplemented samples was determined based on our previous study.^24^ Food and water were provided ad libitum, with body weights recorded weekly. At the end of the study, mice were euthanized via CO_2_ asphyxiation, and blood was collected for biochemical analysis. The liver, spleen, kidneys, and adipose tissues (perigonadal, retroperitoneal, and mesenteric) were removed, weighed, and photographed. Fecal samples were collected from the colon immediately after the sacrifice. Blood samples were centrifuged at 4000*g* for 15 min at 4 °C, and the supernatant was stored at −80 °C for subsequent analysis. Plasma levels of AST, ALT, uric acid (UA), total cholesterol (TC), triglyceride (TG), LDL, and HDL were measured at the National Laboratory Animal Center (Taipei, Taiwan).

### 2.4 Histopathological Examination

Liver and perigonadal fat tissues were collected, fixed in 10% formalin, and prepared for histological analysis. Paraffin-embedded liver and fat samples were sectioned at 5 μm and stained with hematoxylin and eosin (H&E) to evaluate the cellular morphology. For lipid accumulation assessment, liver tissues (0.5 cm × 0.5 cm) were cryoprotected in 30% sucrose, embedded in optimal cutting temperature (OCT) compound, and sectioned at 8 μm for Oil Red O staining. All stained sections were examined under 200× magnification. Oil Red O staining was performed by the Laboratory Animal Center, National Taiwan University College of Medicine (Taipei, Taiwan). Quantification was performed using ImageJ software on three randomly selected fields of view per section. A minimum of 100 adipocytes per animal were measured to calculate the mean cross-sectional area.

### 2.5 Measurement of Serum Adipokines and Liver Cytokines

Serum leptin and adiponectin levels were measured by using ELISA kits. The Mouse Leptin ELISA Kit (Enzo Life Sciences, Farmingdale, NY) and Mouse Adiponectin ELISA Kit (Abcam, Cambridge, U.K.) were used according to the manufacturers’ protocols. Serum samples were diluted appropriately (8× for leptin, 4000× for adiponectin) and incubated in 96-well plates along with standards and detection antibodies. After washing, substrate solution was added, followed by a stop solution, and absorbance was recorded at 450 nm. Hormone concentrations were calculated using standard curves. Additionally, hepatic cytokine levels of tumor necrosis factor-α (TNF-α), interleukin-6 (IL-6), and interleukin-10 (IL-10) were quantified using the Mouse TNF-α, IL-6, and IL-10 Uncoated ELISA Kit (Elabscience, Houston, TX). Liver homogenates were prepared, and the supernatants were collected for analysis. Capture antibodies were coated onto plates that were incubated overnight at 4 °C. After blocking, 100 μL of standards or samples was added and incubated for 1 h. Detection antibodies and Avidin-HRP were then applied sequentially, each followed by incubation and washing steps. The reaction was developed using a TMB substrate and stopped with sulfuric acid. The absorbance was measured at 450 nm, and cytokine concentrations were determined by using standard curves.

### 2.6 Glucose Metabolism Assessment

Fasting blood glucose (FBG) was measured after an 8-h fast, during which food was removed, bedding was changed, and fructose solution was replaced with distilled water. Blood samples were taken from the tail vein and applied to glucose test strips for measurement using a glucometer. Serum insulin levels were quantified using the Mouse Insulin ELISA Kit (Mercodia, Uppsala, Sweden). After the 8-h fast, blood was also collected via cardiac puncture using a heparin-coated syringe and then centrifuged at 3500*g* for 15 min at 4 °C. The resulting supernatant was collected, centrifuged again, and stored at −80 °C until analysis. Standards and serum samples (10 μL) were added to a 96-well plate with enzyme conjugate solution and incubated at room temperature (20–25 °C) for 2 h. After the sample was washed, the TMB substrate was added, and it was incubated in the dark for 15 min. The absorbance was measured at 450 nm, and insulin concentrations were determined by using a standard curve. HOMA-IR was calculated from fasting glucose and insulin values. Oral glucose tolerance tests (OGTT) were conducted following an 8-h fast with baseline glucose levels recorded at 0 min, followed by oral gavage of a 40% glucose solution at a dose of 2 g/kg body weight. Tail vein blood was collected at 30, 60, 90, and 120 min, and glucose levels were measured by using a glucometer.

### 2.7 Western Blot Analysis

Liver tissues were homogenized in gold lysis buffer supplemented with protease inhibitors to extract the total protein. The lysates were centrifuged at 12,000*g* for 1 h at 4 °C, and protein concentrations were measured using the Bio-Rad protein assay (Bio-Rad Laboratories). Equal amounts of protein (25 μg) were combined with 5× sample buffer, heated at 100 °C for 10 min, and separated by SDS-PAGE using 10, 12, or 13.5% gels at 50 mA. Proteins were transferred onto PVDF membranes with transfer buffer (25 mM Tris-HCl, 192 mM glycine, and 20% methanol) and then blocked with 1% bovine serum albumin in 20 mM Tris-HCl buffer for 1.5 h. Membranes were incubated with primary antibodies, washed with TPBS, and then incubated with HRP-conjugated secondary antibodies (diluted 1:2500 or 1:5000) for 1 h at room temperature (20–25 °C). Protein bands were detected by using enhanced chemiluminescence (ECL) and quantified by using ImageJ software. Prior to normalization, the stability of GAPDH expression across all experimental groups was verified to ensure its suitability as a loading control, consistent with validated protocols in similar obesity models,^25,26^ with no significant variations observed between the ND and HFFD groups.

### 2.8 Short-Chain Fatty Acid (SCFA) Analysis

SCFA concentrations in cecal content were quantified using gas chromatography–mass spectrometry (GC–MS) following the previous method.^27^ Briefly, 100 mg of fecal sample was homogenized in 0.5% phosphoric acid, extracted with ethyl acetate (containing 4-methylvaleric acid as the internal standard), and centrifuged at 18,000*g*. The supernatant was analyzed using an Agilent GC-MS system equipped with a DB-WAXetr capillary column (30 m × 0.25 mm, 0.25 μm). Compounds were identified using the NIST library and quantified based on standard calibration curves. In accordance with the validated protocol, the method exhibited high linearity (*R* ^2^ > 0.999), with limits of detection (LOD) ranging from 0.49 to 1.50 μM and intraday/interday precision (RSD) maintained below 6% ensuring robust quantification of acetate, propionate, and butyrate.

### 2.9 Gut Microbiota Analysis

Total genomic DNA was extracted from cecal content using the innuPREP Stool DNA Kit (IST Innuscreen, Berlin, Germany) following the manufacturer’s protocol. The hypervariable V3–V4 region of the bacterial 16S rRNA gene was amplified using the universal primers 341F (5′-CCTACGGGNGGCWGCAG-3′) and 805R (5′-GACTACHVGGGTATCTAATCC-3′). PCR amplicons were purified, quantified, and sequenced on an Illumina MiSeq platform (Illumina, San Diego, CA) to generate paired-end reads (2 × 300 bp), targeting a sequencing depth of >30,000 reads per sample at Biotools Co., Ltd. (Taipei, Taiwan). Bioinformatics analysis was conducted using the QIIME 2 pipeline. Raw sequences were quality-filtered, denoised, merged, and chimera-removed using the DADA2 plugin to generate amplicon sequence variants (ASVs). Taxonomic classification was assigned to representative ASVs against the SILVA database. α diversity indices (Chao1 and Shannon) and β diversity metrics (Principal Coordinate Analysis based on UniFrac distances) were calculated to evaluate microbial community structure.

### 2.10 Statistical Analysis

Data are expressed as means ± the standard error of the mean (SEM). Statistical analyses were carried out using SPSS Statistics 22.0. One-way ANOVA was performed, followed by Duncan’s multiple range test for post hoc comparisons. A *p*-value less than 0.05 was considered statistically significant. Graphs were created by using GraphPad Prism.

## 3 Results

### 3.1 Fermentation Enhances the Bioactive Profile of Black Soybean and Dehulled Adlay

The bioactive composition and antioxidant capacity of FBA were characterized to evaluate the effects of fermentation. Fermentation by *B. subtilis* *natto* significantly enhanced the bioactive profile compared to UFBA. Specifically, as illustrated in Figure 1a, the DPPH radical scavenging activity increased significantly from 13.0 ± 1.03% in UFBA to 26.1 ± 4.15% in FBA. Consistent with this improved antioxidant potential, Figure 1b demonstrates that the total phenolic content rose from 23.1 ± 0.03 mg GAE/g in UFBA to 29.1 ± 0.15 mg GAE/g in FBA. Most notably, as shown in Figure 1c, γ-polyglutamic acid (γ-PGA), a functional metabolite absent in the unfermented substrate, was synthesized *de novo* at a concentration of 8.8 ± 0.38 mg/g in FBA. These findings indicate that the fermentation process successfully enriched key bioactive compounds, particularly polyphenols and γ-PGA.

**Figure 1:**
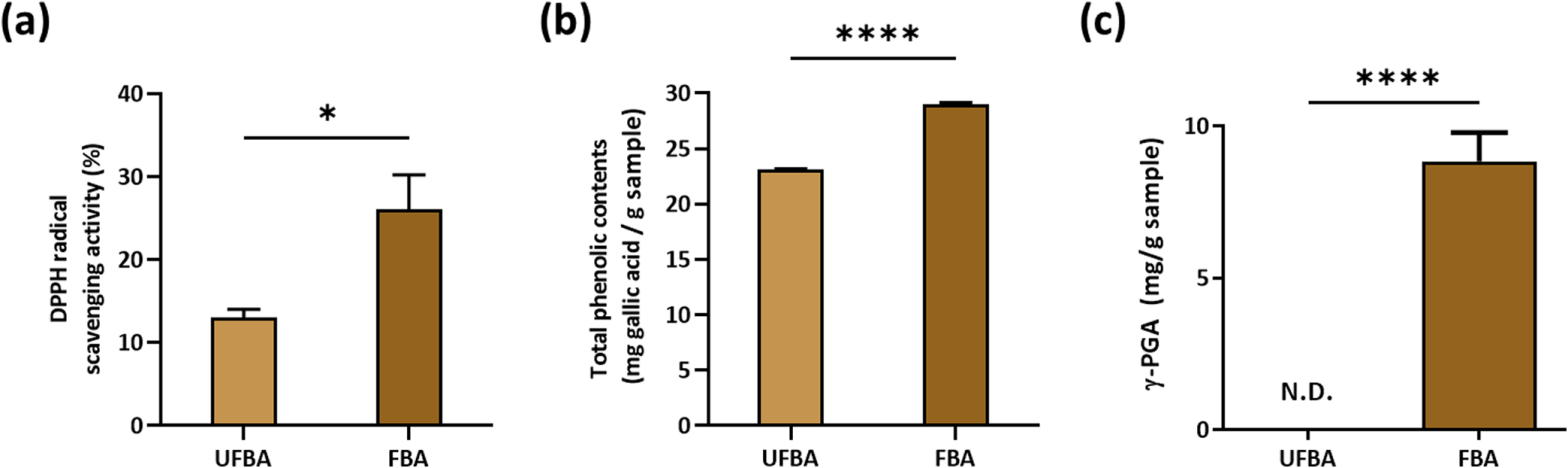
Bioactive composition and antioxidant activity of unfermented and fermented black soybean and dehulled adlay. (a) DPPH radical scavenging activity (%). (b) Total phenolic contents expressed as mg gallic acid equivalents (GAE)/g sample. (c) γ-polyglutamic acid (γ-PGA) concentration. UFBA: Unfermented black soybean and dehulled adlay; FBA:*B. subtilis* *natto*-fermented black soybean and dehulled adlay. Data are presented as mean ± SEM (*n* = 3). Statistical significance was analyzed using Student’s *t* test (*p < 0.05, ****p < 0.0001). N.D.: Not detected.

### 3.2 FBA Supplementation Suppresses HFFD-Induced Weight Gain

The experimental design and treatment timeline are illustrated in Figure 2a. To ensure that the observed metabolic effects were not attributed to differences in caloric intake, the daily consumption of both a solid diet and fructose water was monitored. As detailed in Table 1, the ND group had a higher food intake compared to the HFFD group (ND: 3.61 ± 0.05 g/day; HFFD: 3.12 ± 0.15 g/day; UFBA: 3.29 ± 0.14 g/day; FBA: 3.01 ± 0.07 g/day), this is attributed to the lower energy density of the standard diet. Regarding total energy input, the ND group had a significantly lower daily caloric intake (12.08 ± 0.18 kcal/day) compared with the HFFD-fed groups. Crucially, there were no significant differences in total daily caloric intake among the HFFD (17.89 ± 0.64 kcal/day), UFBA (17.91 ± 0.60 kcal/day), and FBA (17.34 ± 0.35 kcal/day) groups. This isocaloric profile confirms that the superior antiobesity efficacy observed in the FBA group was not driven by reduced energy consumption but rather by the metabolic regulatory properties of the fermented intervention. Given the 6% dietary inclusion rate, this was selected to approximate a realistic human consumption level. Based on the body surface area normalization method, this dosage corresponds to a human equivalent dose of approximately 29.3 g per day for a 60 kg adult, which represents a feasible daily serving size for a functional food intervention. Metabolic syndrome is strongly linked to increased body weight as obesity is a key risk factor in its development. Based on the gross morphological examination at week 15, as shown in Figure 2b, the HFFD-fed mice exhibited a distinct obese phenotype compared to the lean ND group. This morphological change was consistent with the significant increase in body weight observed in the HFFD group. As illustrated in Figure 2c, FBA significantly inhibited weight gain starting from week 8 (FBA: 30.68 ± 0.92 g; HFFD: 33.94 ± 0.90 g) and persisted until the end of the study (FBA: 39.30 ± 1.61 g; HFFD: 44.88 ± 1.66 g), whereas UFBA supplementation failed to prevent significant weight gain.

**Figure 2:**
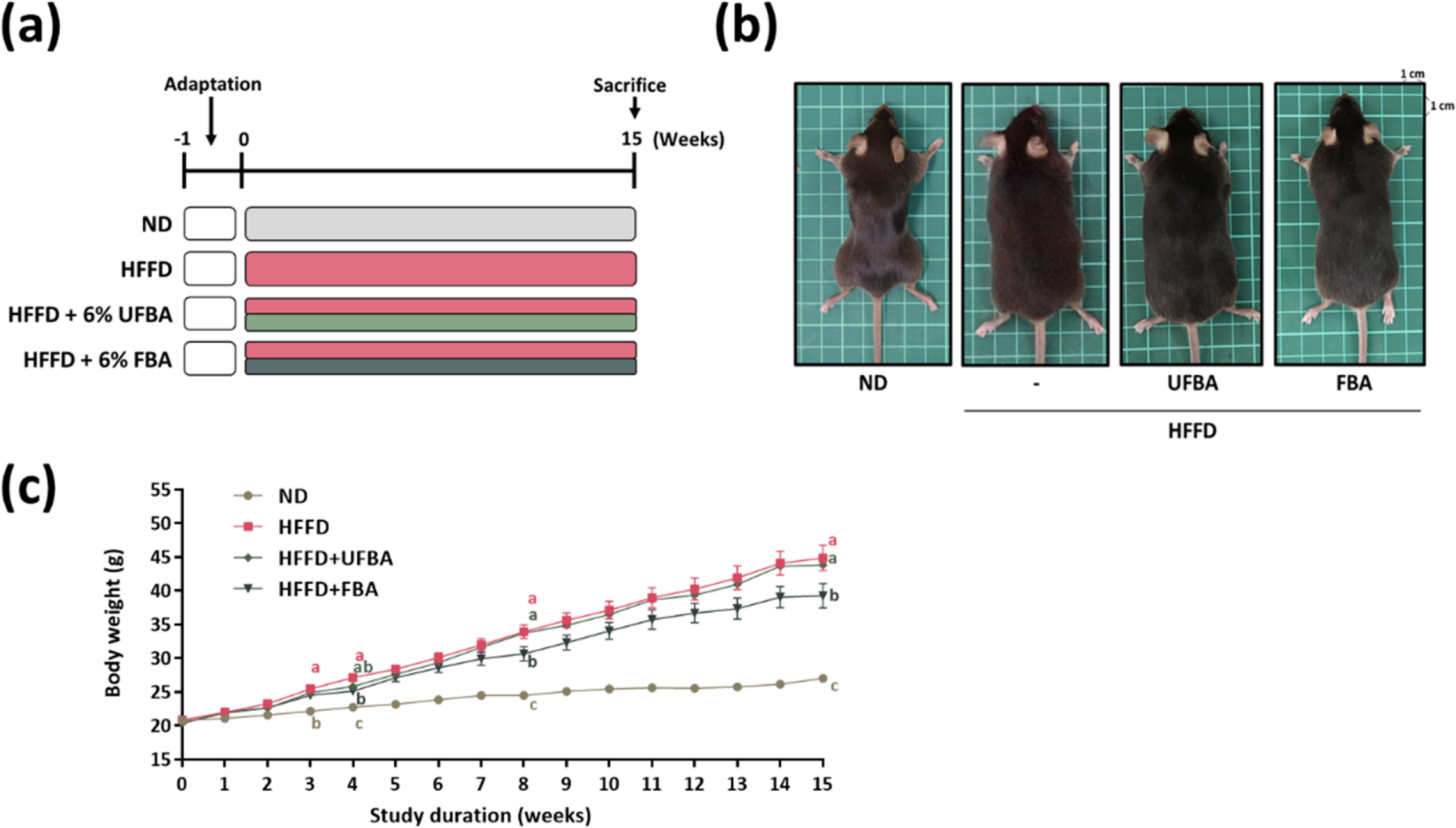
FBA supplementation reduced the body weight gain in HFFD-fed C57BL/6 mice. (a) Schematic of the experimental design. (b) Photographs of mice from each experimental group at the end of week 15. (c) Weekly tracking of the average body weight of each group expressed as means ± SEM (*n* = 8). Statistical significance (*p* < 0.05) is indicated by different letters (a–c) for each respective group.

**Table 1:** Dietary and Drinking Calories Calculation for HFFD-Induced C57BL/6J Mice^a^

	ND	HFFD	UFBA	FBA
food intake (g/day)	3.61 ± 0.05^a^	3.12 ± 0.15^b^	3.29 ± 0.14^ab^	3.01 ± 0.07^b^
food calories (kcal/day)	12.08 ± 0.18^b^	14.73 ± 0.69^a^	15.34 ± 0.67^a^	14.19 ± 0.67^a^
water intake (mL/day)	4.77 ± 0.10^a^	3.82 ± 0.09^b^	3.40 ± 0.10^c^	3.75 ± 0.14^b^
fructose calories (kcal/day)		3.05 ± 0.07^a^	2.72 ± 0.08^b^	3.00 ± 0.11^a^
total calories (kcal/day)	12.08 ± 0.18^b^	17.89 ± 0.64^a^	17.91 ± 0.60^a^	17.34 ± 0.35^a^

a Data was expressed as means ± SEM (*n* = 8). The significance difference was analyzed by using one-way ANOVA and Duncan’s Multiple Comparison test. Values with different letters (a–c) are significantly different (*p* < 0.05) between each group.

### 3.3 FBA Supplementation Improves Serum Biochemical Parameters by Reducing Liver Enzymes, Lipids, and Uric Acid Levels in HFFD-Fed Mice

The serum biochemical parameters presented in Table 2 indicate that the HFFD group had significantly elevated AST (307.91 ± 39.50 U/L) and ALT (96.76 ± 27.78 U/L) levels compared to those of the ND group (AST: 78.67 ± 6.06 U/L; ALT: 25.53 ± 1.25 U/L). Both UFBA and FBA supplementation improved these markers, with FBA showing a significant reduction in AST (UFBA - AST: 252.77 ± 36.99 U/L; ALT: 74.03 ± 13.36 U/L; FBA - AST: 177.87 ± 44.40 U/L; ALT: 47.21 ± 6.70 U/L). Additionally, UA levels were higher in the HFFD group (3.56 ± 0.31 mg/dL) but improved with UFBA (2.91 ± 0.22 mg/dL) and were significantly reduced by FBA (2.52 ± 0.41 mg/dL). The HFFD group showed significantly higher T-CHO (233.9 ± 25.92 mg/dL) and TG (106.26 ± 14.92 mg/dL) levels compared to the ND group (T-CHO: 88.41 ± 4.67 mg/dL; TG: 48.79 ± 4.73 mg/dL). FBA significantly decreased T-CHO levels (202.39 ± 40.45 mg/dL), whereas UFBA did not show a similar effect (234.64 ± 19.36 mg/dL). Both UFBA (69.61 ± 7.62 mg/dL) and FBA (68.81 ± 5.71 mg/dL) significantly reduced TG levels, with FBA achieving the greatest improvement. Additionally, LDL-C levels were significantly elevated in the HFFD group (41.81 ± 2.87 mg/dL) but were significantly lowered by FBA (32.02 ± 2.93 mg/dL), while UFBA (39.40 ± 2.82 mg/dL) had no significant impact. The T-CHO/HDL-C and LDL-C/HDL-C ratios were significantly higher in the HFFD group (1.36 ± 0.03 and 0.24 ± 0.01) compared to the ND group (1.19 ± 0.01 and 0.12 ± 0.01, respectively). FBA supplementation significantly decreased the LDL-C/HDL-C (0.18 ± 0.03) compared to the HFFD group, whereas no significant difference was observed in the T-CHO/HDL-C (1.32 ± 0.01).

**Table 2:** FBA Supplementation Improved the Serum Biochemical Parameters in HFFD-Fed C57BL/6 Mice^a^

	ND	HFFD	UFBA	FBA
AST (U/L)	78.67 ± 6.06^c^	307.91 ± 39.50^a^	252.77 ± 36.99^ab^	177.87 ± 44.40^bc^
ALT (U/L)	25.53 ± 1.25^b^	96.76 ± 29.78^a^	74.03 ± 13.36^ab^	47.21 ± 6.70^ab^
UA (mg/dL)	3.26 ± 0.22^ab^	3.56 ± 0.31^a^	2.91 ± 0.22^ab^	2.52 ± 0.41^b^
T-CHO (mg/dL)	88.41 ± 4.67^c^	233.90 ± 25.92^a^	234.64 ± 19.36^a^	202.39 ± 40.45^b^
TG (mg/dL)	48.79 ± 4.73^b^	106.26 ± 14.92^a^	69.61 ± 7.62^b^	68.81 ± 5.71^b^
HDL-C (mg/dL)	74.36 ± 1.72^b^	172.04 ± 3.59^a^	169.90 ± 3.86^a^	153.96 ± 11.70^a^
LDL-C (mg/dL)	8.99 ± 0.82^c^	41.81 ± 2.87^a^	39.40 ± 2.82^a^	32.02 ± 2.93^b^
T-CHO/HDL	1.19 ± 0.01^c^	1.36 ± 0.03^a,b^	1.38 ± 0.02^a^	1.32 ± 0.01^b^
LDL/HDL	0.12 ± 0.01^c^	0.24 ± 0.01^a^	0.23 ± 0.01^a,b^	0.18 ± 0.03^b^

a Data are expressed as means ± SEM (*n* = 7–8). The significance of the difference was analyzed by one-way ANOVA and Duncan’s Multiple Comparison test. Values with different letters (a–e) are significantly different (*p* < 0.05) between each group.

### 3.4 FBA Supplementation Reduces Liver Enlargement, Organ Weights, and Adipose Tissue Accumulation in HFFD-Fed Mice

As shown in Figure 3a, livers in the HFFD group were larger, pinkish, and exhibited visible oil droplets, while those in the ND group appeared to be more reddish. Following UFBA and FBA supplementation, liver size decreased, and the color became more similar to that of the ND group. Organ weight analysis revealed a descending order for liver, kidneys, and spleen weights across groups: HFFD, UFBA, FBA, and ND. Specifically, liver and kidney weights were significantly higher in the HFFD group (liver: 1.99 ± 0.17 g; kidneys 0.41 ± 0.01 g) compared to ND (liver: 1.18 ± 0.13 g; kidneys 0.35 ± 0.01 g), while FBA supplementation significantly reduced kidney weight (liver: 1.51 ± 0.12 g; kidneys 0.37 ± 0.01 g). The results were consistent with the elevated serum ALT and AST levels. We also calculated the organ indices (organ weight normalized to body weight), as illustrated in Figure S1. However, due to the disproportionate increase in body weight driven by adipose tissue expansion in the HFFD group, the liver index did not show statistically significant differences among groups. As shown in Figure 4, the lack of difference in the relative index, despite the observable hepatomegaly and steatosis, suggests that absolute organ weight serves as a more accurate indicator of pathological hypertrophy in this obesity model. As illustrated in Figure 3b, white adipose tissue (WAT), the combined weight of perigonadal (pWAT), retroperitoneal (rWAT), and mesenteric (mWAT) fat, was markedly larger in the HFFD group (4.4 ± 0.10 g) than in the ND group (0.44 ± 0.39 g). Supplementation with UFBA (3.69 ± 0.32 g) and FBA (3.1 ± 0.41 g) reduced the total fat mass, with FBA producing the most pronounced decrease. rWAT and mWAT were lower in UFBA (pWAT: 2.28 ± 0.04 g; rWAT: 0.94 ± 0.09 g; mWAT: 0.70 ± 0.08 g) compared to HFFD (pWAT: 2.30 ± 0.05 g; rWAT: 1.21 ± 0.07 g; mWAT: 0.93 ± 0.09 g), while FBA (pWAT: 1.73 ± 0.25 g; rWAT: 0.79 ± 0.10 g; mWAT: 0.44 ± 0.09 g) had the lowest weights for pWAT, rWAT, and mWAT.

**Figure 3:**
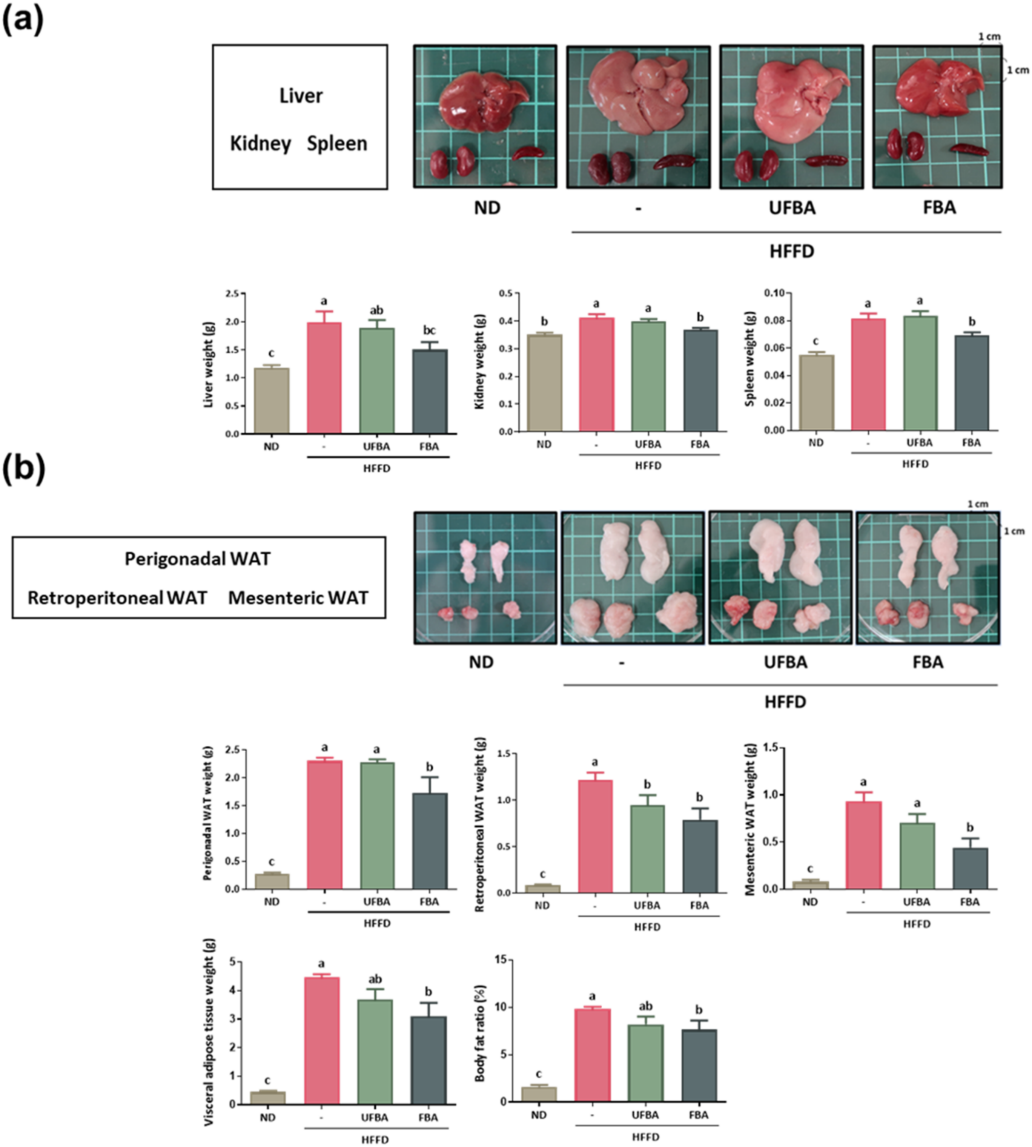
FBA supplementation reduced organ weight gain and visceral adipose tissue mass in HFFD-fed C57BL/6J mice. (a) Images of liver, kidneys, and spleen specimens, accompanied by bar graphs depicting their respective weights. (b) Images of perigonadal, retroperitoneal, and mesenteric adipose tissues, complemented by bar graphs showing their weights. The body fat ratio (%) was determined by dividing the combined weight of visceral adipose tissues (perigonadal + retroperitoneal + mesenteric fat) by the final body weight and multiplying by 100. Statistical data are expressed as mean ± SEM (*n* = 8), with different letters (a–c) denoting statistically significant differences (*p* < 0.05) among groups.

**Figure 4:**
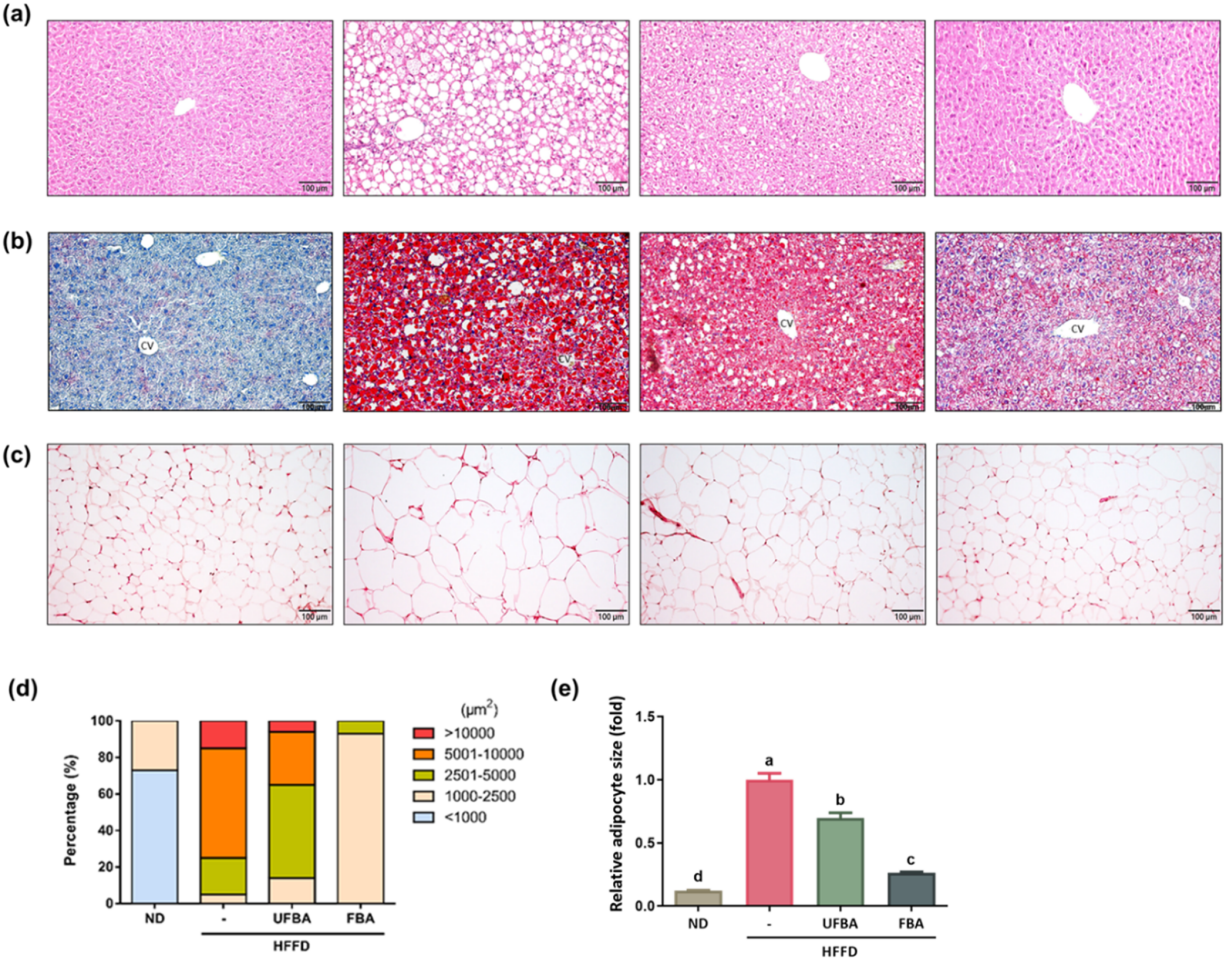
FBA supplementation reduces hepatic lipid accumulation and adipocyte size in HFFD-fed C57BL/6 mice. (a) Histological examination of liver tissue using hematoxylin and eosin (H&E) staining. (b) Lipid-specific staining of liver sections with Oil Red O. Liver specimens underwent standard histological processing: fixation, dehydration, embedding, and sectioning at 5-μm thickness, followed by staining with H&E and Oil Red O. Images were acquired at 200× magnification for each experimental group. Note: “CV” denotes the central vein. (c) Histological images of adipose tissue sections (magnification: 200×) for each experimental group. (d) Analysis of adipocyte size distribution. (e) Quantitative assessment of relative adipocyte size using ImageJ software on representative histological sections. Statistical data are presented as means ± SEM (*n* = 4). Different letters (a–c) indicate statistically significant differences between groups (*p* < 0.05).

### 3.5 FBA Supplementation Reduces HFFD-Induced Hepatic Fat Accumulation and White Adipose Tissue Size

The liver H&E results in Figure 4a reveal that, compared to the ND group, the HFFD group exhibited shrunken hepatocyte nuclei, disorganized cellular arrangement, and infiltration of inflammatory cells. There was a significant increase in macrovesicles, indicating hepatic lipid accumulation caused by the HFFD. Supplementation with both UFBA and FBA reduced the number of fat vacuoles compared to HFFD. In Figure 4b, liver Oil Red O staining shows that ND livers had tightly arranged hepatocytes with few red-stained fat particles, whereas HFFD livers displayed greater fat accumulation, resulting in a redder tissue appearance. UFBA and FBA supplementation lessened fat accumulation relative to HFFD. The adipose tissue H&E results in Figure 4c, observed at 200× magnification, show that the HFFD group had fewer but notably larger adipocytes compared to ND. In the UFBA and FBA groups, adipocytes were smaller with a greater number of cells visible in the same field, with FBA demonstrating the most pronounced improvement. Figure 4d indicates that the HFFD group exhibited profound hypertrophy, with 60% of adipocytes ranging from 5001 to 10,000 μm^2^ and 15% exceeding 10,000 μm^2^. Supplementation with UFBA and FBA markedly altered this distribution pattern. Specifically, UFBA supplementation shifted the population primarily toward an intermediate size, with 51% of cells falling within the 2501–5000 μm^2^ range. In contrast, FBA treatment resulted in a more substantial reduction in cell size, with 93% of adipocytes concentrated in the 1000–2500 μm^2^ range. As shown in Figure 4e, FBA supplementation caused a 3.7-fold reduction in the average relative adipocyte size compared with the HFFD group. These quantitative findings indicate that FBA mitigates adipocyte hypertrophy, consistent with the observed reduction in visceral fat mass.

### 3.6 FBA Supplementation Improved Glucose Tolerance in HFFD-Fed Mice

As shown in Figure 5a, by week 8, fasting blood glucose levels were significantly higher in the HFFD group (152.63 ± 5.40 mg/dL) compared to those in the ND group (99.63 ± 1.49 mg/dL), indicating hyperglycemia development. Supplementation with UFBA (127.38 ± 3.64 mg/dL) and FBA (117.50 ± 6.05 mg/dL) effectively reduced fasting blood glucose levels, with FBA showing a slightly greater reduction. At the end of the study, glucose levels remained significantly elevated in HFFD (204.50 ± 8.72 mg/dL) versus ND (116.13 ± 3.25 mg/dL). UFBA (188.25 ± 9.68 mg/dL) showed a downward trend without a significant difference, whereas FBA (164.75 ± 4.32 mg/dL) resulted in a significant decrease. As shown in Figure 5b, OGTT results reveal that blood glucose levels were consistently lower in the ND group than in the HFFD group. After glucose administration, levels peaked at 30 min and then gradually decreased, with the ND group showing the most substantial decline between 30 and 60 min. In contrast, the HFFD group maintained significantly elevated glucose levels at all measured time points. Figure 5c shows the OGTT area under the curve (AUC), which was significantly lower in the ND group (26,112.86 ± 1140.50 mg/dL) than in the HFFD group (43,497.86 ± 1599.67 mg/dL), indicating impaired glucose tolerance in the HFFD group. Notably, FBA supplementation (37,030.71 ± 1739.76 mg/dL) significantly reduced the AUC, indicating improved glucose regulation. Figure 5d shows that serum insulin levels were markedly elevated in the HFFD group (6.65 ± 1.21 μg/L) compared to those in the ND group (1.07 ± 0.18 μg/L), while FBA supplementation (2.84 ± 0.64 μg/L) significantly lowered these levels. Similarly, HOMA-IR values in Figure 5e indicated that insulin resistance was significantly higher in the HFFD group (3.6 ± 0.87) than in the ND group (0.33 ± 0.06), with a decreasing trend after FBA supplementation (1.21 ± 0.25).

**Figure 5:**
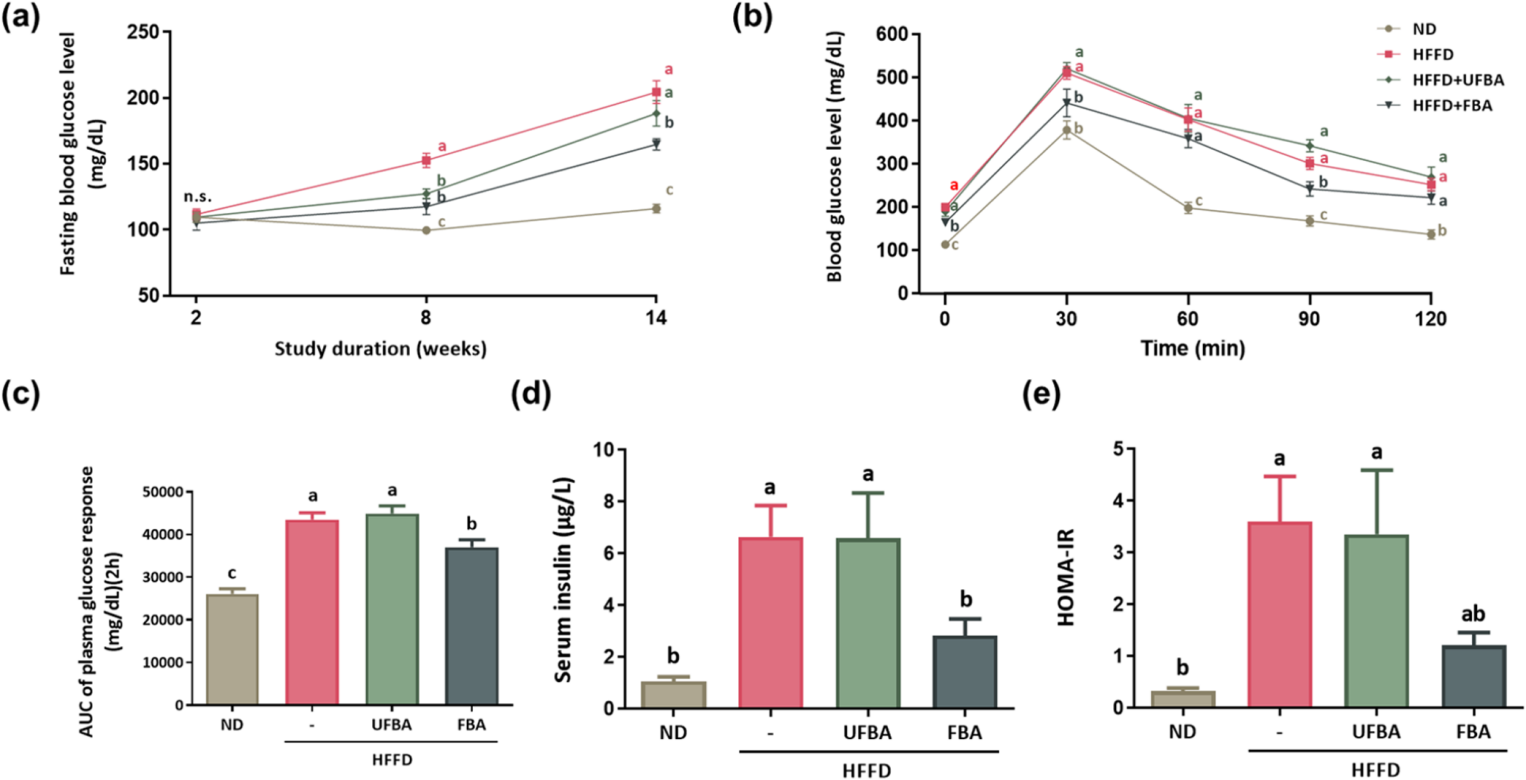
FBA supplementation improves glucose tolerance in HFFD-fed C57BL/6 mice. (a) Fasting blood glucose levels were measured at weeks 2, 8, and 15 using a glucose meter. (b) Glycemic response curves obtained from the oral glucose tolerance test (OGTT). (c) Corresponding area under the curve (AUC) values. Mice were fasted for 10 h prior to blood collection. (d) Serum insulin concentrations were determined using a commercial assay kit. (e) Homeostatic model assessment of insulin resistance (HOMA-IR). Data are presented as means ± SEM (*n* = 7–8). Different letters (a–c) indicate statistically significant differences between groups (*p* < 0.05).

### 3.7 FBA Modulates Adipokine Balance and Inflammatory Responses

Metabolic disorders caused by an HFFD are strongly linked to adipokine imbalance and chronic inflammation, both contributing to obesity-related complications. Figure 6a shows that serum leptin levels were significantly elevated in the HFFD group (20.2 ± 1.88 ng/mL) compared to those in the ND group (0.87 ± 0.08 ng/mL). UFBA supplementation (17.19 ± 1.44 ng/mL) showed a decreasing trend, while FBA supplementation (11.24 ± 3.38 ng/mL) significantly lowered levels of leptin. Figure 6b indicates that serum adiponectin levels were significantly reduced in the HFFD group (1304.17 ± 35.58 pg/mL) compared to those in the ND group (1900.0 ± 311.23 pg/mL). While UFBA (1349.17 ± 80.78 pg/mL) and FBA (1536.67 ± 84.17 pg/mL) supplementation showed no significant effect, Figure 6c demonstrates that the leptin/adiponectin ratio was significantly elevated in the HFFD group (15.21 ± 1.14) but significantly improved with FBA supplementation (7.27 ± 2.59). Figure 6d,e display the liver inflammation markers, revealing significantly higher TNF-α and IL-6 levels in the HFFD group (TNF-α: 15.22 ± 2.11 pg/μg; IL-6:0.62 ± 0.15 pg/μg) compared to the ND group (TNF-α: 10.06 ± 0.94 pg/μg; IL-6:0.25 ± 0.02 pg/μg). UFBA supplementation (TNF-α: 12.65 ± 0.81 pg/μg; IL-6:0.53 ± 0.11 pg/μg) showed a decreasing trend in these markers, and FBA supplementation (TNF-α: 9.51 ± 0.79 pg/μg; IL-6:0.30 ± 0.04 pg/μg) resulted in a significant decrease in TNF-α and IL-6 levels. IL-10, an anti-inflammatory cytokine essential for regulating immune responses and reducing inflammation, was significantly lower in the HFFD group (4.09 ± 0.38 pg/μg) compared to the ND group (7.94 ± 0.78 pg/μg), as shown in Figure 6f. Both UFBA (6.89 ± 0.38 pg/μg) and FBA (7.73 ± 0.69 pg/μg) supplementation significantly elevated IL-10 levels, with FBA producing the most notable increase.

**Figure 6:**
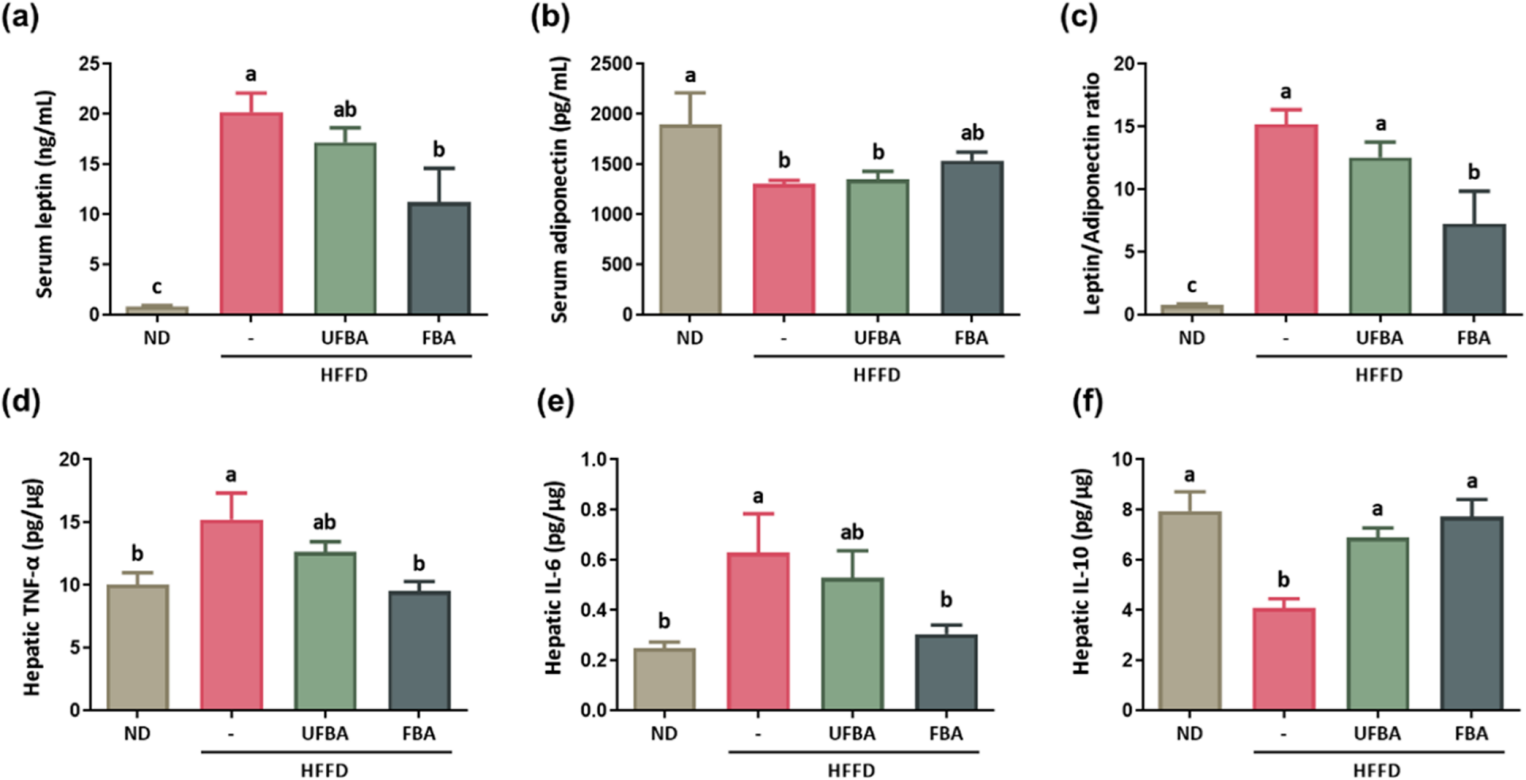
FBA supplementation improves serum leptin levels and modulates liver inflammatory cytokines in HFFD-fed C57BL/6 mice. (a) Serum leptin and (b) serum adiponectin levels were measured using a commercial kit. (c) The leptin-to-adiponectin ratio was calculated. Data are presented as means ± SEM (*n* = 6–7). Quantification of liver cytokines: (d) TNF-α, (e) IL-6, and (f) IL-10 levels were measured using a commercial kit. Data are presented as means ± SEM (*n* = 4–6). Different letters (a–c) indicate statistically significant differences between groups (*p* < 0.05).

### 3.8 FBA Supplementation Activates the AMPK–SIRT1 Signaling Pathway in the Liver and Adipose Tissue of HFFD-Fed Mice

Figure 7a shows that pAMPK/AMPK levels were significantly suppressed in the HFFD group, whereas FBA supplementation considerably increased their level of expression. Protein analysis related to hepatic lipid synthesis and fatty acid β-oxidation indicated that both UFBA and FBA significantly elevated pACC/ACC levels and reduced FASN expression compared to the HFFD group. SCD expression decreased in the UFBA group and was significantly lower in the FBA group, contributing to reduced lipid synthesis. Additionally, Figure 7b illustrates the impact of FBA on enhancing the fatty acid oxidation pathways. Both UFBA and FBA significantly increased SIRT1 expression, with FBA additionally upregulating PGC-1α and CPT-1A, thus promoting fatty acid oxidation metabolism. These results indicate that FBA supplementation improves hepatic lipid metabolism by activating the AMPK–SIRT1 signaling pathway.

**Figure 7:**
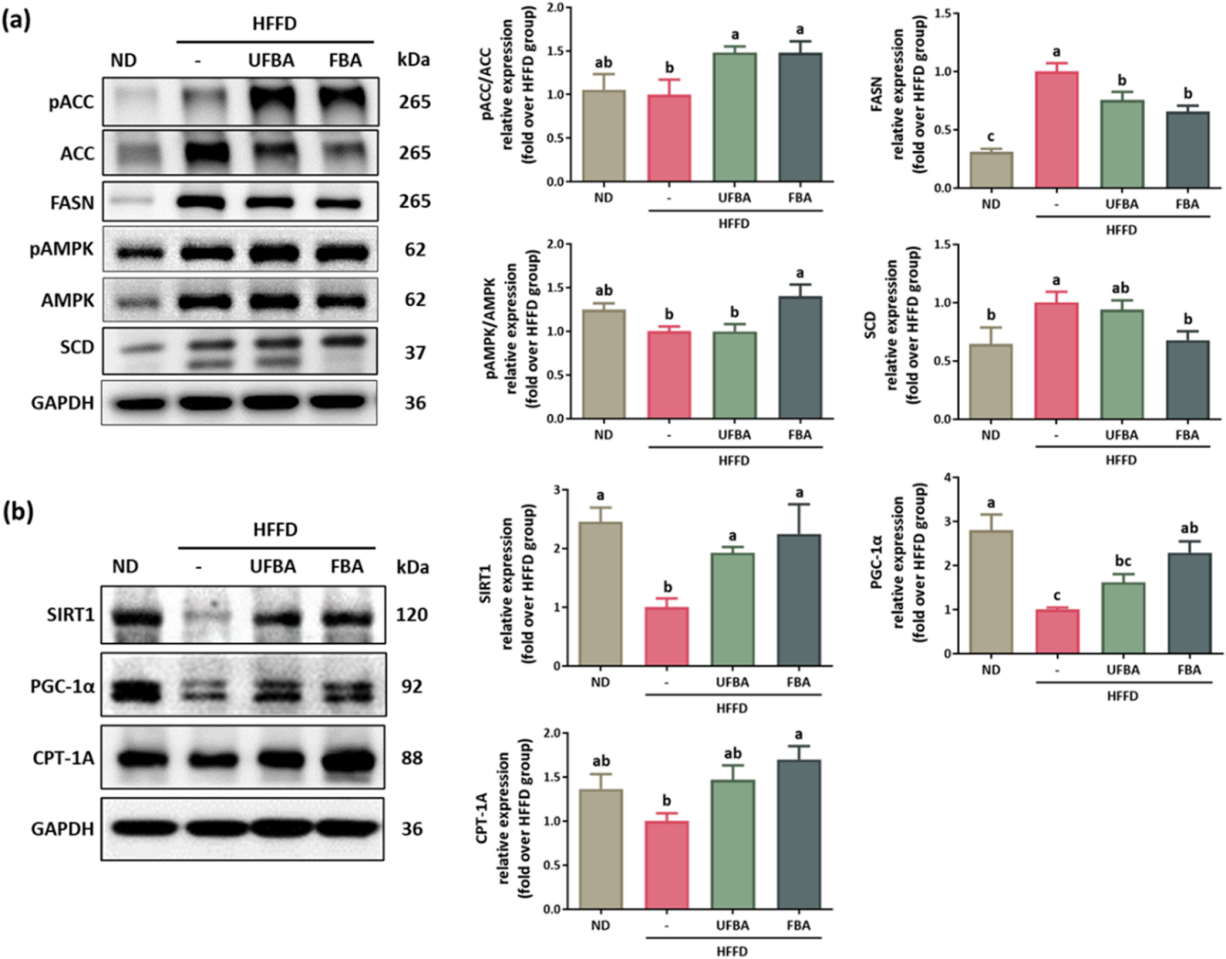
FBA supplementation regulates hepatic AMPK–SIRT1 signaling in HFFD-fed C57BL/6 mice. (a) Western blot analysis of protein levels of pACC (Ser79), ACC, FASN, pAMPK (Thr172), AMPK, and SCD associated with lipogenesis, as quantified using ImageJ software. (b) Western blot analysis of proteins involved in fatty acid oxidation: SIRT1, PGC-1α, and CPT-1A, with subsequent quantification using ImageJ. Statistical data are presented as means ± SEM (*n* = 4). Different letters (a–c) indicate statistically significant differences between groups (*p* < 0.05).

Beyond the liver, AMPK activation in adipose tissue plays a crucial role in lipid metabolism. Figure 8 shows that FBA supplementation significantly increased the pAMPK/AMPK ratio in the adipose tissue. Analysis of lipid metabolism markers demonstrated that both UFBA and FBA significantly elevated pACC/ACC levels, with FBA exerting the most pronounced effect in reducing lipid synthesis. Regarding fatty acid oxidation, FBA notably upregulated PGC-1α expression, thus enhancing fatty acid oxidation metabolism. These results indicate that FBA supplementation effectively activates AMPK signaling in both liver and adipose tissue, leading to improved lipid metabolism and increased fatty acid oxidation in HFFD-fed mice.

**Figure 8:**
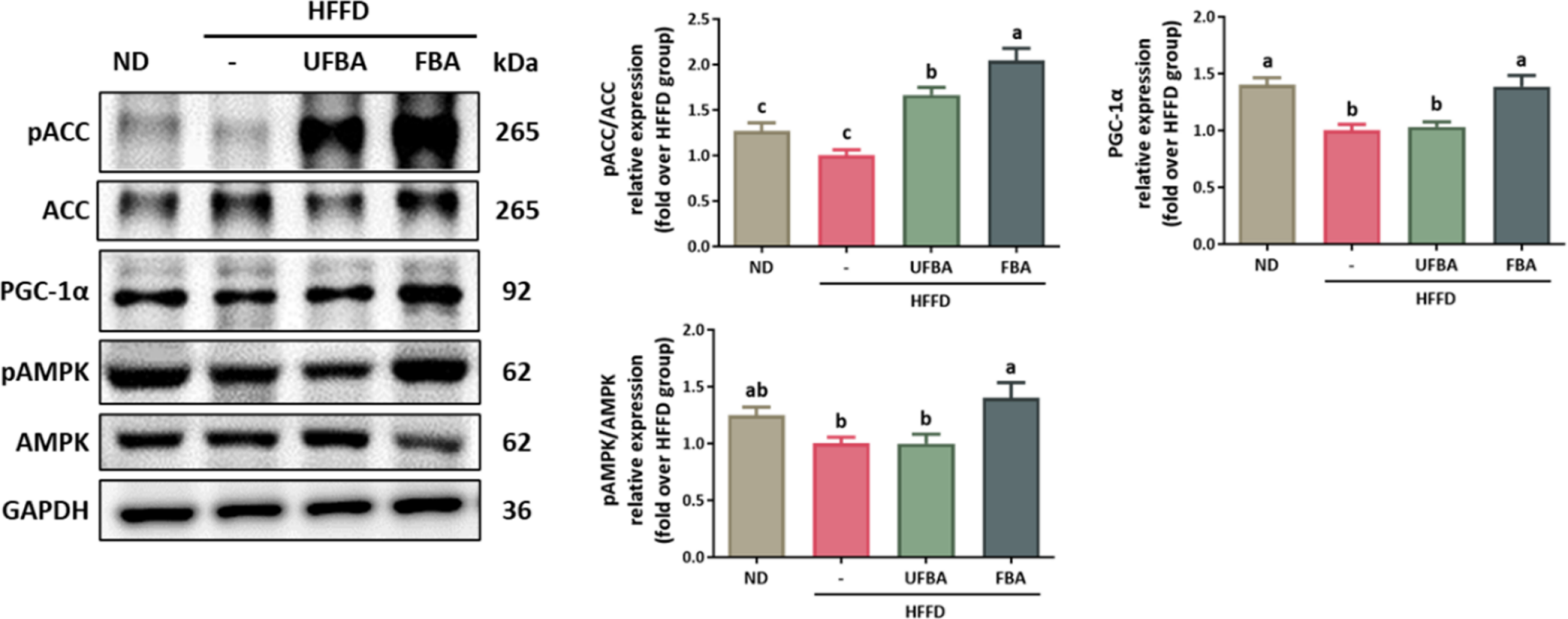
FBA supplementation regulates AMPK signaling in adipose tissue of HFFD-fed C57BL/6 mice. Western blot analysis was conducted to determine protein expression levels of pACC (Ser79), ACC, PGC-1α, pAMPK (Thr172), and AMPK in adipose tissue samples. Subsequent quantification of the results was performed using ImageJ software. Statistical data are presented as means ± SEM (*n* = 4). Different letters (a–c) denote statistically significant differences between groups (*p* < 0.05).

### 3.9 FBA Supplementation Modulates Gut Microbiota Diversity, Composition, and Short-Chain Fatty Acid Levels in HFFD-Fed Mice

Gut microbiota diversity serves as a crucial indicator of microbial ecosystem stability and has a major impact on metabolic health. Figure 9a,9b demonstrates that α diversity, which reflects species richness and evenness within a sample, decreased in the HFFD group compared with the ND group. After supplementation with UFBA and FBA, diversity indices showed a rising trend. β diversity, which represents differences in microbial composition between groups, was assessed by using PLS-DA, as shown in Figure 9c. The microbial profile of the HFFD group differed markedly from that of the ND group, while FBA supplementation led to a distinct new microbial distribution, suggesting its influence on gut microbiota composition. Figure 9d,g shows shifts in microbial communities with an increased Firmicutes-to-Bacteroidetes (F/B) ratio in the HFFD group, commonly associated with metabolic disorders. FBA supplementation induced a downward trend in this ratio. At the class and genus levels, Figure 9e,9f reveals that the HFFD group had a higher relative abundance of *Erysipelotrichia* and the genera *Faecalibaculum*, *Lactobacillus*, and *Dubosiella*, all of which declined following UFBA and FBA supplementation. In contrast, *Bacteroidia* and the genera *Muribaculum*, *Paramuribaculum*, and *Bacteroides* were decreased in the HFFD group but increased after supplementation. Consistent with findings from previous study,^28–30^ an HFFD altered gut microbiota composition by promoting the growth of *Faecalibaculum*, *Erysipelotrichaceae*, *Actinobacteria*, *Bacilli*, and *Lactobacilli*. In contrast, the FBA group showed *Kineothrix* as the most abundant genus, while levels of *Clostridium*, *Lachnospiraceae*, and *Eubacteriales* were restored to those comparable to those of the ND group, indicating that FBA supplementation helps reestablish microbial balance. Figure 9h–9j illustrates the impact of supplementation on short-chain fatty acid (SCFA) levels, which are key gut-derived metabolites involved in host metabolism. Notably, the HFFD group had significantly reduced cecal acetate concentrations compared to those of the ND group, and there were no significant changes in acetate levels following either UFBA or FBA supplementation. Propionate levels were reduced in the HFFD group but increased with UFBA and showed a significant rise with FBA. Similarly, butyrate levels were significantly lower in the HFFD group, remained unchanged after UFBA supplementation, and exhibited a nonsignificant upward trend with FBA. These results indicate that although FBA affects gut microbiota composition, its influence on SCFA production requires further investigation.

**Figure 9:**
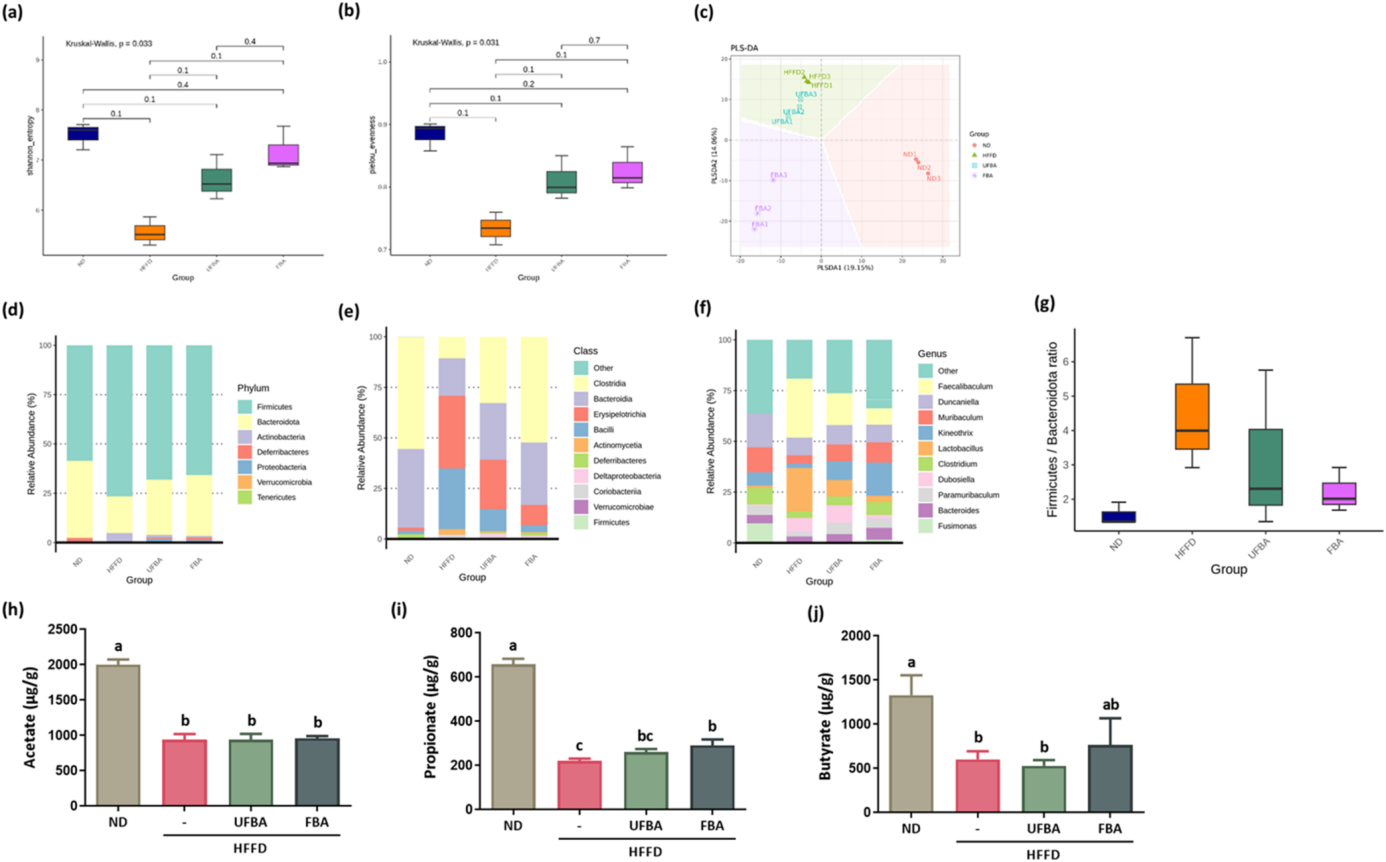
FBA supplementation modulates gut microbiota composition and increases propionate levels in HFFD-fed C57BL/6 mice. (a) Microbial community diversity was evaluated using the Shannon diversity index (α diversity) and (b) Pielou’s evenness index. Box plots depict the median, interquartile range, minimum, and maximum values (*n* = 3 per group). (c) Microbial profiling of fecal samples was conducted using next-generation sequencing (*n* = 3 per group), followed by multivariate analysis with partial least-squares discriminant analysis (PLS-DA) for clustering. (d–g) Relative abundance analysis of the predominant gut bacterial taxa (top 10) at the phylum level, (e) class, and (f) genus levels, complemented by (g) the Firmicutes/Bacteroidetes ratio analysis. (h–j) Concentrations of short-chain fatty acids (SCFAs) were measured in cecal fecal samples via GC–MS, specifically quantifying (h) acetate, (i) propionate, and (j) butyrate levels. Data are presented as means ± SEM (*n* = 4). Statistical significance was assessed using one-way ANOVA followed by Duncan’s multiple comparison test, with different letters (a–c) indicating significant intergroup differences (*p* < 0.05).

## 4 Discussion

Metabolic syndrome is a multifactorial condition resulting from complex interactions among the diet, inflammation, insulin resistance, lipid metabolism, and gut microbiota. Although dietary interventions are commonly used to manage metabolic disorders, effective treatment often requires approaches targeting multiple pathways.^31^ Male C57BL/6J mice were specifically selected for this investigation due to their greater susceptibility to diet-induced metabolic syndrome compared to females^32,33^ and the more pronounced visceral fat reduction observed in males in our previous study.^24^ This study shows that cofermented black soybean and dehulled adlay (FBA) with *B. subtilis* *natto* provides metabolic benefits, highlighting its potential as a functional food that addresses metabolic syndrome at several physiological levels.^34^ Notably, the FBA group experienced weight loss without a significant change in total daily caloric intake compared with the HFFD group, indicating that its effects arise from metabolic regulation rather than caloric dilution or reduced energy consumption. A critical finding of this study is the superior metabolic efficacy of FBA compared to UFBA. Since both groups share the identical substrate matrix and thus a similar profile of basal macronutrients and fibers, this indicates the essential role of fermentation. Our quantitative analysis points to fermentation-derived bioactives as the key drivers. Specifically, we identified the *de novo* synthesis of γ-PGA (8.8 mg/g) and a significant enrichment of total phenolics (29.1 mg GAE/g) in FBA, which were either absent or lower in UFBA. These findings indicate that the bioconversion process significantly potentiates the metabolic regulatory capacity of the black soybean and adlay substrate. This is consistent with recent research indicating that polyphenol-rich foods,^35^ especially fermented products, can influence lipid metabolism by boosting fat oxidation and reducing adipogenesis.^36–38^ The modulation of the AMPK–SIRT1 signaling pathway in the liver and adipose tissue provides a plausible molecular basis for these observed metabolic improvements. AMPK is a crucial energy sensor that enhances energy expenditure.^39–41^ The concurrent upregulation of AMPK phosphorylation and SIRT1 expression observed in the liver and adipose tissue is consistent with their well-established functional interplay in regulating cellular energy homeostasis.^42^ The activation of AMPK is known to lead to phosphorylation of ACC at Ser79. This specific modification inhibits ACC activity and reduces malonyl-CoA production, thereby acting as a critical molecular switch to suppress lipogenesis.^43,44^ The reduction in malonyl-CoA relieves the allosteric inhibition of CPT-1A, which is universally recognized as the rate-limiting enzyme for mitochondrial fatty acid oxidation.^45,46^ Consequently, the observed upregulation of both pACC and CPT-1A in our study, coupled with the significant reduction in visceral fat and hepatic steatosis, suggests that FBA contributes to enhanced fatty acid oxidation *in vivo*. While direct enzymatic activity assays were not performed, the coherence between these signaling markers and the ameliorated lipid phenotypes strongly supports the involvement of this pathway. Regarding glucose homeostasis, the upregulation of PI3K/Akt phosphorylation suggests enhanced hepatic insulin signaling. It is important to distinguish whether improved insulin sensitivity is a primary effect of the intervention or is secondary to weight loss. In our study, while the UFBA group exhibited reduced adiposity, it failed to significantly lower HOMA-IR. In contrast, FBA treatment robustly improved HOMA-IR and insulin signaling markers. The reduction in pro-inflammatory cytokines (TNF-α, IL-6) alongside the increase in IL-10 underscores the role of FBA in modulating the immune response.^47,48^ Chronic low-grade inflammation is a defining feature of metabolic syndrome, driven by adipose tissue dysfunction and immune cell infiltration.^49^ FBA’s ability to restore adiponectin levels is especially important, as this adipokine enhances insulin sensitivity and provides anti-inflammatory effects.^50^ The bioactive compounds in FBA, such as polyphenols, bioactive peptides, γ-PGA, and microbial metabolites, likely contribute to these immunomodulatory actions. Fermentation not only increases the bioavailability of these compounds but also induces the production of γ-PGA, a microbial polymer synthesized by *B. subtilis* *natto*, which has been shown to improve lipid metabolism, lower serum triglyceride levels, and regulate immune responses.^9,47,51^ Moreover, γ-PGA exhibits prebiotic-like effects that support gut barrier function and promote the growth of beneficial microbes. For example, γ-PGA treatment in a loperamide-induced constipation mouse model significantly upregulated the expression of tight junction proteins, reduced inflammatory markers, and restored intestinal barrier integrity.^52^ Consistent with these findings, gut microbiota analysis in this study revealed a reduced Firmicutes/Bacteroidetes ratio in the FBA group, suggesting a shift toward a healthier microbial composition that is associated with improved metabolic outcomes.^53–55^ Although the causal validity of this ratio is currently debated, in the context of our study, it indicates a reversal of the phenotype enriched in the obese phenotype. Specifically, this structural restoration coincided with the reduction of *Faecalibaculum* and *Dubosiella*.^56^ While *Faecalibaculum* is known as an SCFA producer, its expansion is frequently reported in HFFD-induced dysbiosis models, where it facilitates the rapid production and immediate host absorption of SCFAs to lipogenesis.^29,30^ This mechanism explains the discordance between the high abundance of *Faecalibaculum* and the reduced fecal SCFA levels observed in the HFFD group; thus, its suppression by FBA likely reflects a recovery from dysbiosis. Furthermore, we observed that *Kineothrix* became the most predominant genus in the FBA group. *Kineothrix* is widely documented as a butyrate-producing taxon,^57,58^ and its proliferation may contribute to the maintenance of the gut environment. Regarding short-chain fatty acids (SCFAs), our results revealed a nuanced profile rather than a generalized increase. Cecal acetate levels remained suppressed in the HFFD-fed groups, and butyrate showed only a nonsignificant increasing trend despite the abundance of butyrate-producing taxa, which may reflect rapid utilization by colonocytes or cross-feeding interactions. However, crucially, FBA treatment resulted in a significant elevation of propionate. Physiologically, propionate is the primary SCFA that reaches the liver via the portal vein, where it acts as a key regulator of gluconeogenesis and lipid metabolism.^59,60^ This specific elevation of propionate aligns with the observed improvements in hepatic insulin sensitivity and reduced lipogenesis in the FBA group. Our current microbiome analysis is based on 16S compositional data; future studies utilizing functional metagenomics or fecal microbiota transplantation (FMT) are warranted to definitively establish causality. In conclusion, this study demonstrates that FBA supplementation alleviates HFFD-induced metabolic syndrome in C57BL/6J mice, exhibiting superior efficacy compared to UFBA. Our data indicate that FBA functions as a multitargeted intervention: it mitigated body weight gain, hepatic steatosis, and systemic inflammation while enhancing insulin sensitivity. These physiological improvements coincided with the modulation of hepatic AMPK–SIRT1 and PI3K–Akt signaling pathways and the restoration of gut microbial balance, characterized specifically by elevated cecal propionate levels. While these findings suggest that fermentation-derived metabolites coordinate a gut-liver regulatory axis, the precise bioactive constituents remain to be fully elucidated. Therefore, future studies integrating untargeted metabolomics with functional metagenomics are warranted to identify the specific active fractions and to definitively establish the causal mechanisms underlying these multisystemic effects.

## Supplementary Material



## Notes

Effect of FBA supplementation on liver, kidney, and spleen indices (Figure S1) (PDF)

## Abbreviations

ACC: acetyl-CoA carboxylase
AMPK: AMP-activated protein kinase
Akt: serine threonine protein kinase
CPT1A: carnitine palmitoyltransferase 1A
FASN: fatty acid synthase
FBA: cofermented black soybean and dehulled adlay
FFA: free fatty acid
FMT: fecal microbiota transplantation
HDL-C: high-density lipoprotein cholesterol
HFFD: high-fat and high-fructose diets
IL-10: interleukin-10
IL-6: interleukin-6
LDL-C: low-density lipoprotein cholesterol
MetS: metabolic syndrome
OGTT: oral glucose tolerance test
PGC-1α: peroxisome proliferator-activated receptor γ coactivator-1α
PI3K: phosphoinositide 3-kinase
PUFA: polyunsaturated fatty acids
SCD: stearoyl-CoA desaturase
SCFAs: short-chain fatty acids
SIRT1: NAD-dependent deacetylase sirtuin-1
TC: total cholesterol
TG: triglyceride
TNF-α: tumor necrosis factor-α
UA: uric acid
UFBA: unfermented black soybean and dehulled adlay
WAT: white adipose tissue
γ-PGA: γ-polyglutamic acid
