# Supplementary material for: Fermented Black Soybean and Dehulled Adlay Improve Metabolic Syndrome via AMPK–SIRT1 Activation and Gut Microbiota Modulation
Source: J Agric Food Chem. 2026 Feb 23;74(8):6864–77. doi: 10.1021/acs.jafc.5c15101 (PMC12964532; doi:10.1021/acs.jafc.5c15101)
Supplement: Supplementary file 1 [file jf5c15101_si_001.pdf]

Supporting Information

Fermented Black Soybean and Dehulled Adlay Improve Metabolic Syndrome via AMPK-SIRT1 Activation and Gut Microbiota Modulation

Ya-Ru Kuo[1], Yi-Wei Zheng[2], Pin-Yu Ho[1], Yi-Chen Lo[1], Po-Jung Tsai[2], Min-Hsiung Pan[1,3,4]*

[1]Institute of Food Sciences and Technology, National Taiwan University, Taipei City 10617, Taiwan

[2]Graduate Program of Nutrition Science, School of Life Science, National Taiwan Normal University, Taipei City 10610, Taiwan

[3]Department of Medical Research, China Medical University Hospital, China Medical University, 40402 Taichung City, Taiwan

[4]Department of Health and Nutrition Biotechnology, Asia University, 41354 Taichung City, Taiwan,

*Please send all correspondence to: Dr. Min-Hsiung Pan

Institute of Food Science and Technology, National Taiwan University

No.1, Section 4, Roosevelt Road, Taipei 10617, Taiwan

Tel: +886-2-33664133; Fax: +886-2-33661771; E-mail: mhpan@ntu.edu.tw

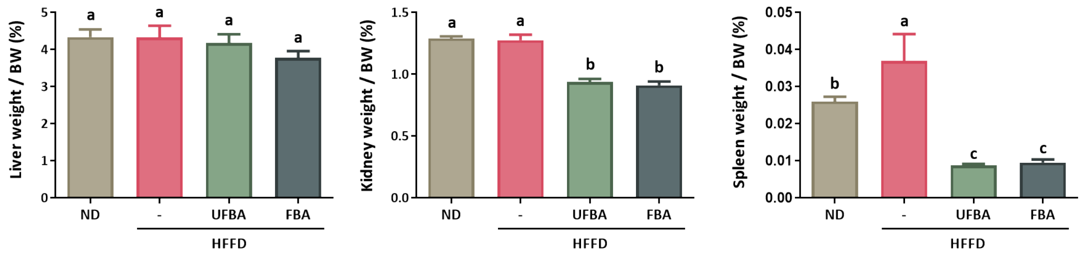

Figure S1. Effect of FBA supplementation on organ indices in HFFD-fed C57BL/6J mice. The relative organ weights were calculated as the ratio of organ weight to final body weight (%) for the liver, kidneys, and spleen. Data are presented as means ± SEM (n = 8). Statistical significance was analyzed using one-way ANOVA followed by Duncan's multiple range test. Different letters (a–c) above the bars indicate statistically significant differences between groups ($p < 0.05$).
